# Differences in the expression of the phosphatase PTP-1B in patients with localized prostate cancer with and without adverse pathological features

**DOI:** 10.3389/fonc.2024.1334845

**Published:** 2024-04-19

**Authors:** Maria T. Bourlon, Shaddai Urbina-Ramirez, Haydee C. Verduzco-Aguirre, Mauricio Mora-Pineda, Hugo E. Velazquez, Eucario Leon-Rodriguez, Yemil Atisha-Fregoso, María G. De Anda-Gonzalez

**Affiliations:** ^1^ Department of Hemato-Oncology, Instituto Nacional de Ciencias Medicas y Nutricion Salvador Zubiran, Mexico City, Mexico; ^2^ Universidad Panamericana, Escuela de Medicina, Mexico City, Mexico; ^3^ Department of Pathology, Instituto Nacional de Ciencias Medicas y Nutricion Salvador Zubiran, Mexico City, Mexico; ^4^ Instituto Nacional de Cardiología “Ignacio Chavez”, Radiology Department, Mexico City, Mexico; ^5^ Instituto Tecnológico de Estudios Superiores de Monterrey, Escuela de Medicina y Ciencias de la Salud, Monterrey, Mexico

**Keywords:** prostate cancer, PTP-1B, prostatectomy, prognostic factors, protein expression

## Abstract

**Introduction:**

Patients with adverse pathological features (APF) at radical prostatectomy (RP) for prostate cancer (PC) are candidates for adjuvant treatment. Clinicians lack reliable markers to predict these APF preoperatively. Protein tyrosine phosphatase 1B (PTP-1B) is involved in migration and invasion of PC, and its expression could predict presence of APF. Our aim was to compare PTP-1B expression in patients with and without APF, and to explore PTP-1B expression as an independent prognostic factor.

**Methods:**

Tissue microarrays (TMAs) were constructed using RP archival specimens for immunohistochemical staining of PTP-1B; expression was reported with a standardized score (0-9). We compared median PTP-1B score between cases with and without APF. We constructed two logistic regression models, one to identify the independence of PTP-1B score from biologically associated variables (metformin use and type 2 diabetes mellitus [T2DM]) and the second to seek independence of known risk factors (Gleason score and prostate specific antigen [PSA]).

**Results:**

A total of 73 specimens were suitable for TMA construction. Forty-four (60%) patients had APF. The median PTP-1B score was higher in those with APF: 8 (5-9) vs 5 (3-8) (p=0.026). In the logistic regression model including T2DM and metformin use, the PTP-1B score maintained statistical significance (OR 1.21, 95% CI 1.01-1.45, p=0.037). In the model including PSA and Gleason score; the PTP-1B score showed no independence (OR 1.68, 95% CI 0.97-1.41, p=0.11). The area under the curve to predict APF for the PTP-1B score was 0.65 (95% CI 0.52-0.78, p=0.03), for PSA+Gleason 0.71 (95% CI 0.59-0.82, p=0.03), and for PSA+Gleason+PTP-1B score 0.73 (95% CI 0.61-0.84, p=0.001).

**Discussion:**

Patients with APF after RP have a higher expression of PTP-1B than those without APF, even after adjusting for T2DM and metformin exposure. PTP-1B has a good accuracy for predicting APF but does not add to known prognostic factors.

## 1 Introduction

Prostate cancer is one of the most common cancers in men worldwide (1), with most cases being diagnosed at early stages. Localized prostate cancer represents a heterogeneous disease exhibiting a broad spectrum of presentation, ranging from asymptomatic disease detected by screening to aggressive tumors with poorer long-term outcomes. Consequently, localized prostate cancer is stratified at diagnosis based on the risk of progression into low-, intermediate- and high-risk disease. This categorization utilizes pretreatment prostate specific antigen (PSA) levels, the Gleason score from the initial biopsy, and the extent of the primary tumor as determined by digital rectal examination (2). Patients with high-risk disease usually require a multimodal approach, combining androgen deprivation therapy with radiotherapy, as prostatectomy alone can be insufficient in those with locally advanced disease. For low- or intermediate-risk patients who are candidates for definitive treatment, surgery is a therapeutic option with a curative intent (3).

Pathological staging may differ from the initial clinical stage, and the final prostatectomy specimen may reveal adverse pathological factors (APF) such as extraprostatic extension, seminal vesicle invasion, or positive surgical margins. Patients with APF are at an increased risk of recurrent disease (4–6), which is frequently managed with adjuvant or salvage radiotherapy (7), which carries an increased probability of significant gastrointestinal and genitourinary toxicity. Even among patients initially classified as low- or intermediate-risk, APFs may be present, suggesting that clinical risk factors alone may not adequately predict the necessity for additional treatment following prostatectomy.

Deregulation of protein tyrosine phosphatases (PTPs) or protein tyrosine kinases (PTKs) leads to aberrant tyrosine phosphorylation, which has been implicated in the etiology of several diseases, including prostate cancer (8–10). The phosphatase PTP-1B, encoded by the PTPN1 gene, plays a role in metabolic signaling pathways, particularly through regulation of the insulin receptor and leptin pathways (11). PTP-1B-deficient mice show increased insulin sensitivity and resistance to obesity (12). This phosphatase also interacts with other receptor protein tyrosine kinases such as PDGFR and EGFR, both *in vitro* and *in vivo (* 13), and plays a role in the activation of the tyrosine kinases c-Src, which are implicated in the development of some types of cancer (14). PTP-1B can also promote apoptosis through down-regulation of pro-survival signaling, and promotion of caspase activity (15).

Despite exhibiting both tumor suppressing and tumor promoting effects, PTPN1 appears to act predominantly as an oncogene. For instance, silencing PTP-1B in breast cancer cell lines is associated with decreased cell proliferation through the negative regulation of HER2 expression (16). However, clinical evidence remains conflicting: expression of PTP-1B has been identified as a favorable prognostic factor for survival in breast cancer (17); overexpression of PTP-1B has also been proposed as a marker for response to chemotherapy in high-grade serous carcinoma (18). Conversely PTP-1B amplification is associated with poorer survival in gastric and colorectal cancer (19, 20).

PTPN1, encoding PTP-1B, has been found to be co-amplified with the androgen receptor (AR) (21). PTP-1B expression can be induced through stimulation of the AR and is associated with nuclear localization of the AR and a higher Ki67 in primary prostate tumors (22). Depletion of PTP-1B delays androgen-dependent tumor growth and alters *in vitro* migration and invasion. PTP-1B also contributes to the neuroendocrine differentiation of prostate cancer (23), associated with a worse prognosis.

Given that the role of PTP-1B in prostate cancer has mostly been explored in preclinical settings, this study aimed to elucidate how PTP-1B expression correlates with adverse prognostic factors in localized disease. The primary objective of this study was to compare the expression of PTP-1B, quantified by immunohistochemistry, in patients with and without adverse pathological factors in prostatectomy specimens. Secondary objectives were to establish a correlation between PTP-1B expression and pre-prostatectomy PSA values, and PTP-1B expression and Gleason score; to estimate overall and cancer-specific survival according to PTP-1B expression; and to evaluate PTP-1B as an independent prognostic factor.

## 2 Materials and methods

### 2.1 Study population

After institutional review board approval, we identified all cases of prostate cancer with a history of radical prostatectomy at our center from January 1990 to December 2015. We included cases with complete clinical and pathological data. We excluded cases that received neoadjuvant therapy, presented evidence of metastatic disease prior to prostatectomy, or lacked available tissue in the pathology archives. Additionally, cases with insufficient tissue for the planned analyses were eliminated.

We collected the following clinical information from both physical and electronic medical records: patient age at the time of surgery, pre-surgical PSA, prior diagnosis and treatment of type 2 diabetes mellitus (T2DM), pathological staging according to the AJCC/TNM version 7.0, Gleason score, and presence of adverse pathological factors as defined by the International Society of Urological Pathology (ISUP). These factors include extraprostatic extension, seminal vesicle invasion, or any positive surgical margins other than apical. Overall survival was calculated from the date of prostatectomy until death from any cause. Cancer-specific survival was calculated from the date of prostatectomy until death from any prostate cancer-related cause.

### 2.2 Sample handling

Upon identification of the prostatectomy specimens, we used hematoxylin and eosin-stained slides to identify representative areas of each specimen. These areas were characterized by the highest Gleason score, and lacked necrosis, inflammation, fibrosis or desmoplasia. Corresponding areas in the paraffin blocks were manually harvested by tru-cut biopsy and placed into a tissue microarray (TMA) base. After constructing the TMA, we reassessed the samples to ensure every sample contained the selected tumor and Gleason score, and a minimum of 30 tumor cells. We ensured every constructed TMA had at least two cases with normal prostate tissue, benign hyperplasia, or intraepithelial prostatic neoplasm to serve as internal controls.

### 2.3 PTP-1B expression by immunohistochemistry

For immunohistochemistry, TMA paraffin blocks were sectioned into 4 μm slides. The last slide of each series was stained with hematoxylin and eosin to ensure the presence of sufficient neoplastic tissue in the TMA blocks. The remaining slides were processed sequentially in xylene and ethanol washes before being submerged in water. Afterwards, citrate buffer was used for heat-induced antigen retrieval, and slides were then washed and rinsed. The mouse monoclonal antibody against PTPN1 (PTP-1B), clone OTI2G3, (formerly known 2G3 [TA503188], OriGene Technologies, Rockville, MD), was applied. The slides were incubated with the primary antibody for 45 minutes at room temperature, then for 10 minutes with a biotin solution, and 10 minutes with an HRP solution. Slides were developed with diaminobenzidine and counterstained with hematoxylin.

Antibody standardization was achieved by comparison with normal breast and prostatic tissue processed with the same immunohistochemistry technique described above. Each sample was tested with antibody dilutions of 1:150, 1:100 and 1:50. The staining with the best quality was selected for the dilution. These were used as external controls for the TMAs.

**Figure 1:**
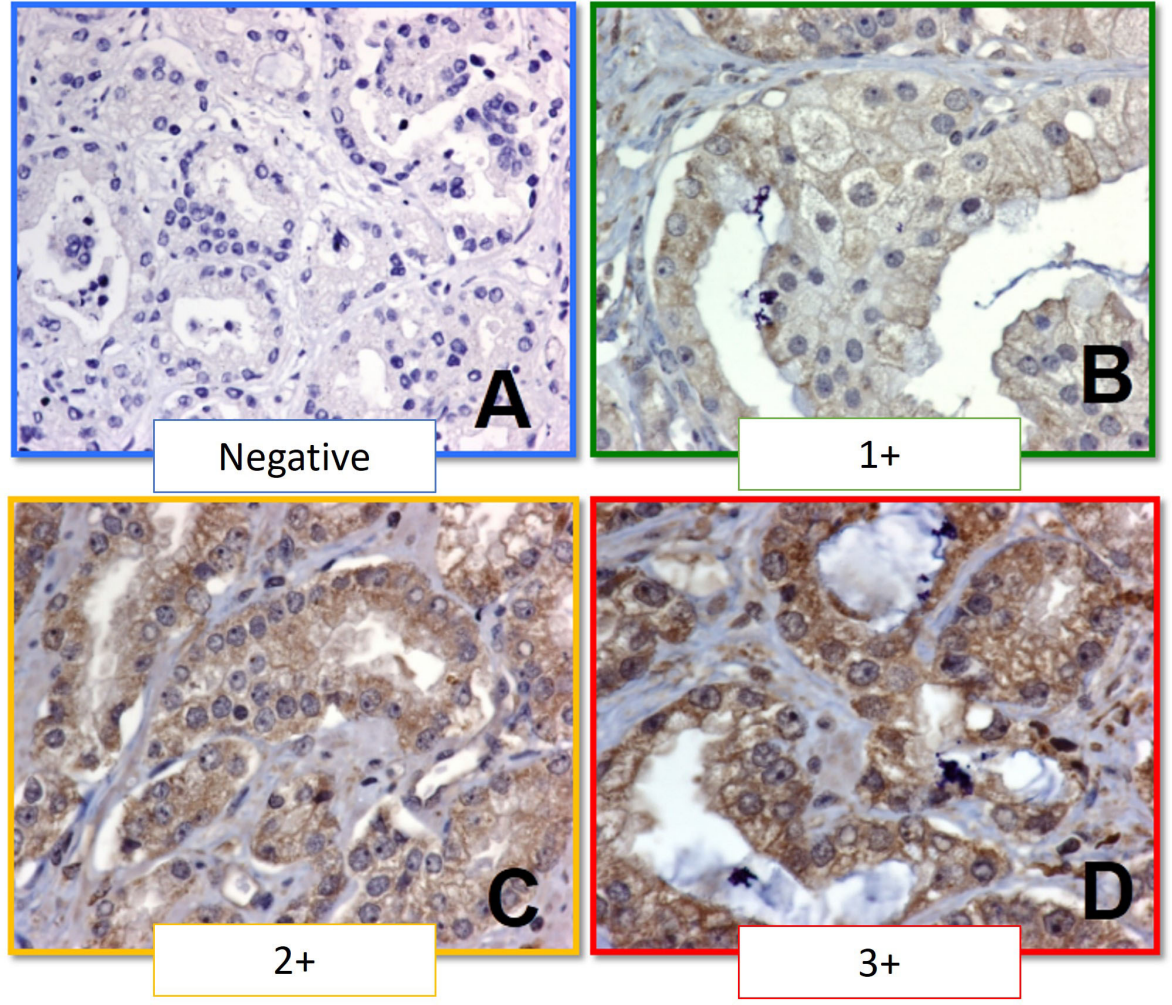
Immunohistochemistry staining scale for PTP-1B. **(A)** Negative (0+). **(B)** Weak staining (1+). **(C)** Moderate staining (2+). **(D)** Strong staining (3+).

To evaluate the antibody in the selected prostate cancer samples, initial examination was conducted at 4x under light microscopy (Nikon Eclipse 80) to ensure antibody expression was visible in a panoramic view. Then, slides were reviewed at 10x and 40x for every TMA cylinder. Antibody expression was evaluated qualitatively as follows: 0, no antibody staining in neoplastic tissue; 1+, weak cytoplasmic staining in neoplastic tissue; 2+ moderate cytoplasmic staining in neoplastic tissue; 3+, strong cytoplasmic staining in neoplastic tissue; NE, not evaluable due to unspecific staining, background staining, no delimitation of malignant cells, or staining of lymphocytes or fibroblasts (**Figure 1**). A second, semi-quantitative assessment categorized staining as: NE; not evaluable, 0, no antibody staining in neoplastic tissue; 1+, 1-50% of staining in neoplastic cells; 2+, 51-75% staining of neoplastic cells; 3+, 76-100% staining of neoplastic cells. An expert pathologist in molecular biology, blinded to clinical data, reviewed the cases. An intensity scale was then created based on the primary and secondary readings, as described by Lessard et al. (22) (**Table 1**).

**Table 1:** PTP-1B staining intensity and final score.

Primary reading	Secondary reading	Final score
0	N/A	0
0	1+, 2+,3 +	1
1+	N/A	2
1+	2+	3
1+	3+	4
2+	1+	5
2+	N/A	6
2+	3+	7
3+	2+	8
3+	N/A	9

N/A, not applicable.

### 2.4 Statistical analysis

The primary aim of the study utilized the Wilcoxon Mann-Whitney test to compare median scores of PTP-1B between groups with and without APF, considering a p-value ≤ 0.05 as statistically significant. Spearman’s test was used for correlation analyses. Logistic regression analysis was employed to examine the association of APF with biologically related variables (metformin use T2DM) and previously identified risk factors (Gleason score and PSA levels). The sensitivity and specificity of the PTP-1B IHC scale to classify prognostic outcomes was determined using 2x2 tables. Receiver operating characteristic (ROC) curves were constructed to identify the optimal cutoff point for the scale. The Kaplan-Meier method was used to estimate overall and cancer-specific survival, with comparisons made using Cox regression analysis.

## 3 Results

### 3.1 Patient characteristics

We identified 502 cases of prostate cancer with a history of radical prostatectomy diagnosed between January 1990 and December 2015 at our center. Of these, 190 pathology specimens were available for review. An expert molecular pathologist eliminated 117 cases due to insufficient material, resulting in 73 cases being included for the construction of TMAs and subsequent PTP-1B IHC analysis.

For the 73 cases included, the median age was 62 years (range 44-78 years). The mean BMI was 27.2 kg/m^2^, with 68.85% of patients classified as overweight or obese. Prior to prostate cancer diagnosis, 20.3% had been diagnosed with T2DM, 21.9% had prior use of metformin (either for T2DM or metabolic syndrome), and 10.95% were on other oral T2DM medications. Regarding clinical characteristics, 38.4% of patients were diagnosed via PSA screening. Most cases (53.4%) had a low Gleason score (6), with approximately one-third of the cases falling into each of the low-, intermediate- and high-risk categories. Population characteristics are further described in **Table 2**.

**Table 2:** Population characteristics (n=73).

Characteristic	N (%)
Age in years (median, range)	62 (44-78)
40-49 years 50-59 years 60-69 years 70 years or older	3 (4.1%) 20 (27.39%) 41 (56.16%) 9 (12.32%)
BMI in kg/m^2^ (mean, SD)	27.2 ± 3.2 (19-32)
Normal (18.5 - 24.9) Overweight (25.0 – 29.9) Obesity (30 or above)	22 (30.13%) 33 (44.2%) 18 (24.65%)
Type 2 diabetes mellitus and medication use	
Prior T2DM diagnosis Metformin Other oral T2DM medications Insulin	15 (20.3%) 16 (21.91%) 8 (10.95%) 1 (1.36%)
Prostate biopsy Gleason score	
6 7 8 9	39 (53.42%) 21 (28.76%) 7 (9.58%) 5 (6.48%)
PSA in ng/mL (median, range)	13.7 (2-84)
Disease risk (D’Amico)	
Low Intermediate High	24 (32.87%) 25 (34.24%) 24 (32.87%)
Pre-prostatectomy clinical stage	
I IIA IIB III	21 (28.76%) 28 (38.35%) 23 (31.5%) 1 (1.36%)

Prostatectomy specimen characteristics are described in **Table 3**. Most patients had tumor sizes occupying less than 50% of the specimen. Gleason scores were 6 in 24.7% of cases, 7 in 40.3% and ≥8 in the remainder. Among these cases, 60.3% presented with at least one adverse pathological factor (APF), such as extraprostatic extension, seminal vesicle invasion, or positive surgical margins. Five cases (6.8%) had pelvic nodal disease.

**Table 3:** Prostatectomy specimen analysis (n=73).

Characteristic	N (%)
Tumor size	
0 - 25% 26 - 50% 51 - 65% 66 - 100%	31 (42.46%) 29 (39.72%) 11 (15.06%) 2 (2.73%)
Gleason score	
6 7 8 9	18 (24.65%) 36 (49.31%) 8 (10.95%) 11 (15.06%)
Adverse pathological factors	
Extraprostatic extension Seminal vesicle invasion Positive surgical margins Any adverse pathological factor	19 (26.02%) 19 (26.02%) 43 (58.90%) 44 (60.27%)
Pelvic lymph node metastases	5 (6.84%)
Clinical stage (AJCC/TNM 7)	
I IIA IIB III IV	6 (8.21%) 13 (17.8%) 30 (41.09% 19 (26.07%) 5 (6.84%)

### 3.2 PTP-1B expression in patients with and without adverse pathological factors

**Figure 2:**
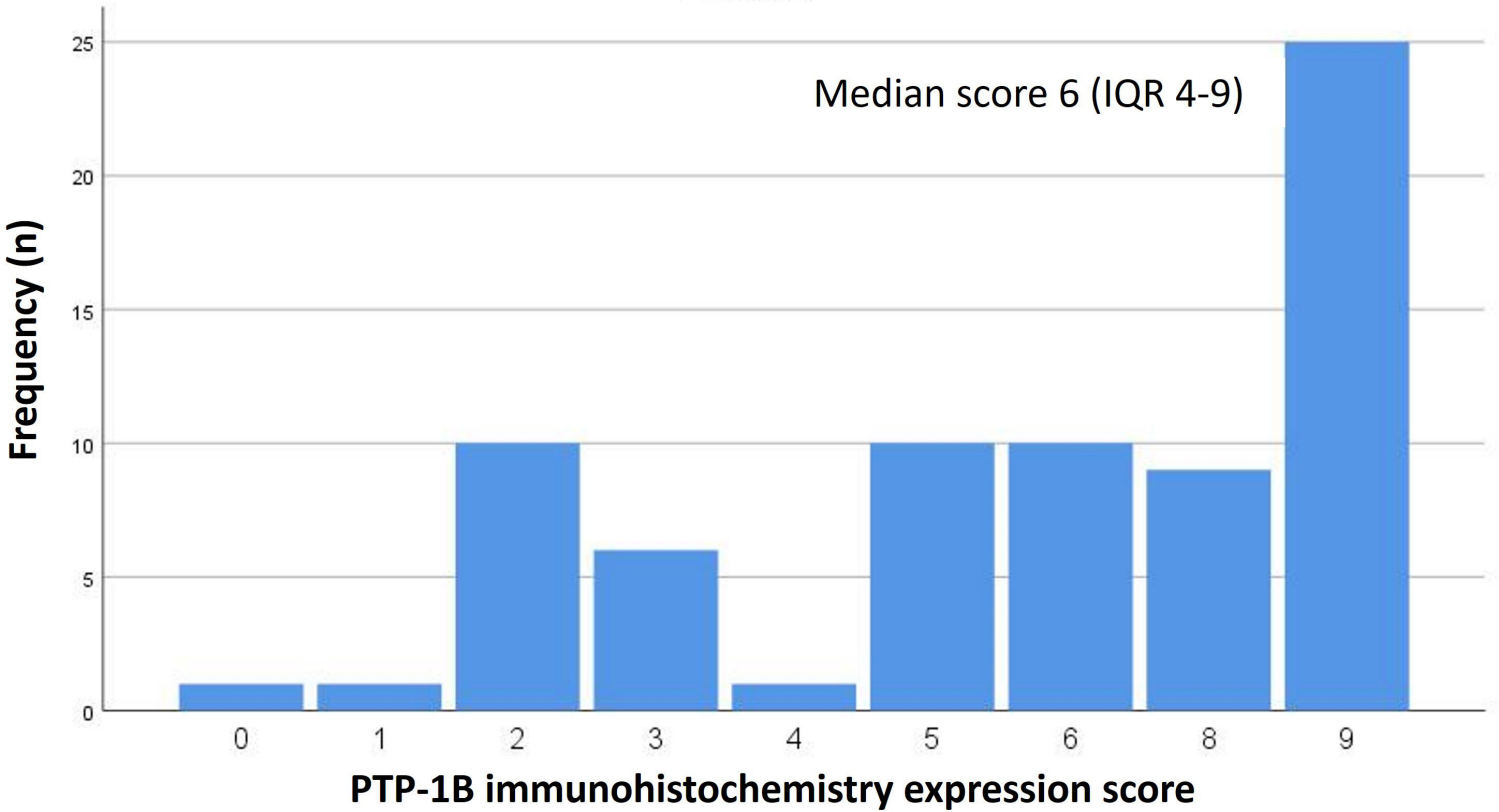
PTP-1B expression score in analyzed prostatectomy specimens.

**Figure 3:**
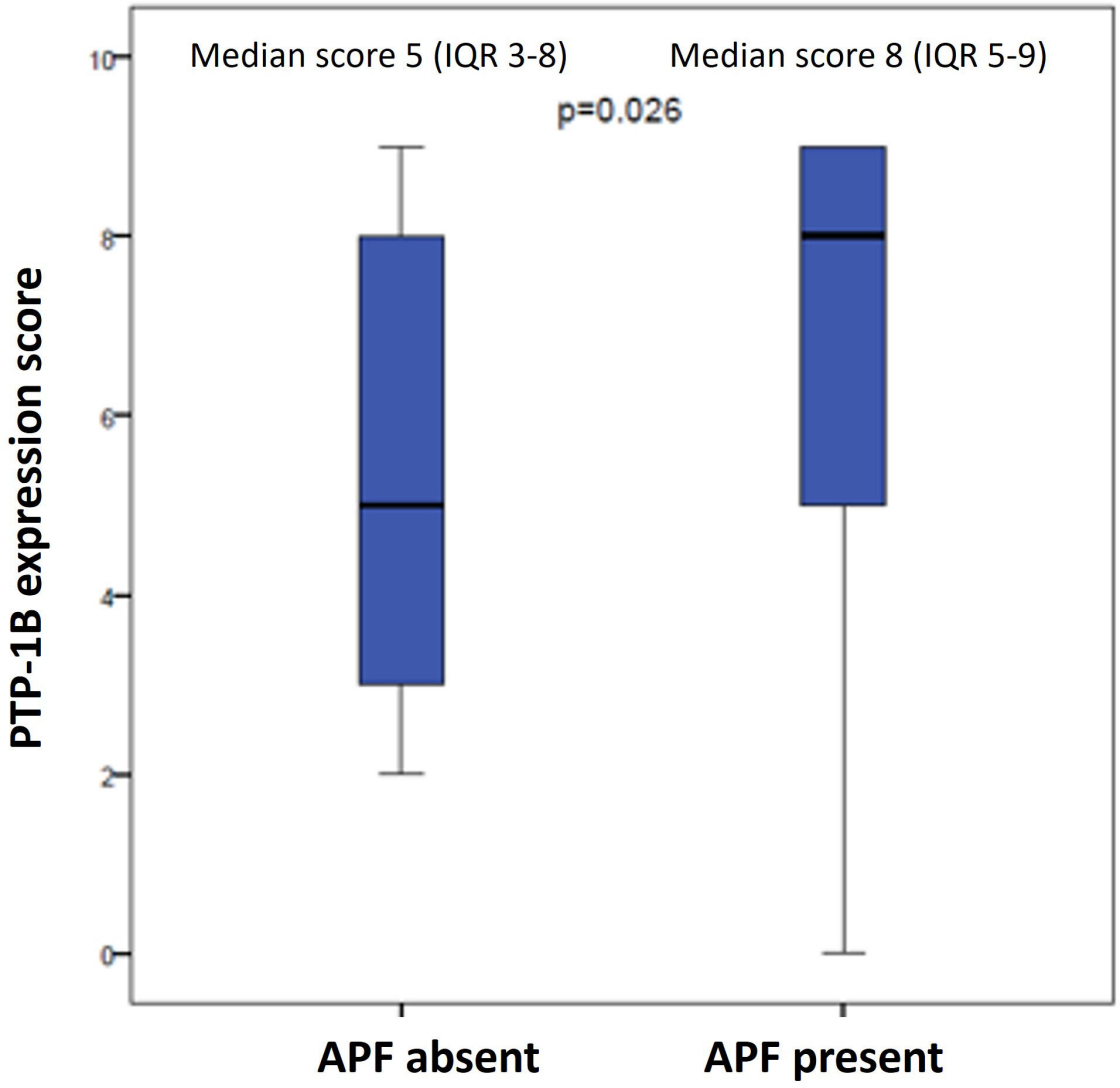
PTP-1B expression score in prostatectomy specimens according to presence or absence of adverse pathological factors (APF).

The median PTP-1B expression score for the entire cohort was 6 (interquartile range [IQR] 4-9) (**Figure 2**). Patients without APFs had a median score of 5 (IQR 3-8), while those with APFs exhibited a significantly higher median score of 8 (IQR 5-9) (p=0.026) (**Figure 3**). A weak correlation was found between PTP-1B expression and both the prostatectomy Gleason score (r=0.24, p=0.042) and the pre-prostatectomy PSA value (r=0.243, p=0.046).

In the logistic regression analysis assessing the association of PTP-1B expression with the presence of APFs, two models were applied. The first model, which included biologically associated variables (history of T2DM and metformin use), found that PTP-1B expression remained significantly associated with the presence of APFs (Odds Ratio [OR] 1.21, 95% Confidence Interval [CI] 1.01-1.45, p=0.037), independent of prior T2DM diagnosis or metformin use. The second model, which incorporated known prognostic factors (PSA and Gleason score), did not find an independent association of PTP-1B score with APFs (OR 1.68, 95% CI 0.97-1.41, p=0.11) (**Table 4**).

**Table 4:** Logistic regression models for association with presence of adverse pathological factors in prostatectomy specimens.

Model 1. Biologically associated variables			
	OR	IC 95%	p
**PTP-1B score**	1.21	1.01-1.45	0.037
**Metformin use**	1.49	0.10-21.77	0.768
**History of T2DM**	0.68	0.45-10.04	0.776
Model 2. Other known prognostic factors			
	**OR**	**IC 95%**	***p***
**PTP-1B score**	1.68	0.97-1.41	0.11
**Gleason score**	1.53	0.83-2.85	0.18
**PSA (ng/ml)**	1.07	0.99-1.15	0.08

**Figure 4:**
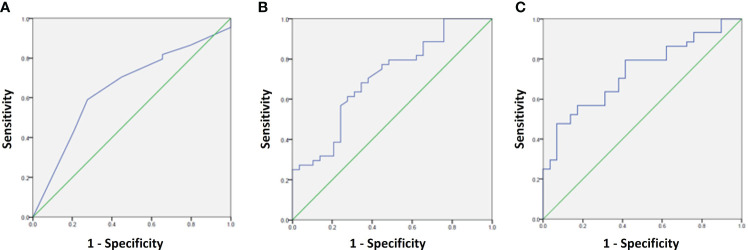
ROC curves for prediction of adverse pathological factors using the following variables: **(A)** PTP-1B score, **(B)** PSA + Gleason score, and **(C)** PSA + Gleason score+ PTP-1B score.

To determine the prognostic utility of the PTP-1B IHC scale, we calculated its sensitivity and specificity in classifying cases with either favorable or unfavorable prognosis using 2x2 tables. Receiver Operating Characteristic (ROC) curves were constructed to select the optimal cut-point for the PTP-1B expression score in predicting the presence of APFs, yielding an Area Under the Curve (AUC) of 0.65 (95% CI 0.52-0.78, p=0.03). A cut-off point of 5 was chosen based on sensitivity and specificity analyses (**Table 5**). Additional ROC curves for PSA and Gleason score demonstrated an AUC of 0.71 (95% CI 0.59-0.82, p=0.03), Combining all predictors (PSA, Gleason score, and PTP-1B score), the AUC was 0.73 (95% CI 0.61-0.84, p=0.001) (**Figure 4**).

**Table 5:** Receiver operating characteristic (ROC) curve for identification of the cut-off point of PTP-1B expression score.

PTP-1B expression score	Sensitivity	Specificity
0.00	1.000	1.000
0.50	0.977	1.000
1.50	0.955	1.000
2.50	0.864	0.793
3.50	0.818	0.793
4.50	0.795	0.655
5.50	0.705	0.448
7.00	0.591	0.276
8.50	0.432	0.207
10.00	0.000	0.000

### 3.3 Survival analysis

The median overall survival (OS) and cancer-specific survival (CSS) for the entire cohort had not been reached, with estimated 15-year OS at 72% and 15-year CSS at 78%, respectively.

**Figure 5:**
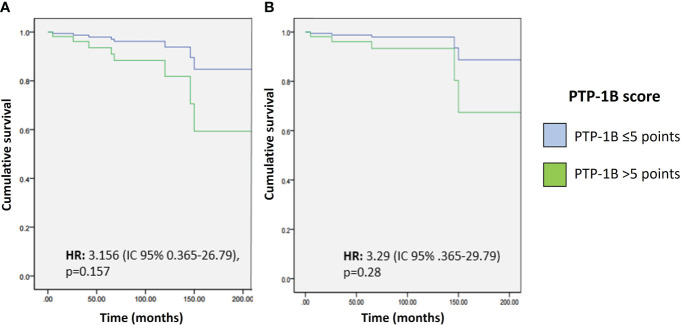
Overall survival **(A)** and cancer-specific survival **(B)** according to PTP-1B score.

Using the previously selected cut-off point, a PTP-1B expression score ≥6 was not significantly associated with OS (HR 3.15, CI 95% 0.36-26.7, p=0.157) or CSS (HR 3.29, CI 95% 0.36-29.73, p=0.288) (**Figure 5**).

## 4 Discussion

The quantification of PTP-1B expression by immunohistochemistry is a low-cost test available at most pathology laboratories. Our study shows that the grade of PTP-1B expression is higher in prostatectomy specimens with adverse pathological factors compared to those without adverse pathological factors. This association persisted even when controlling for prior diagnosis of T2DM and metformin use in logistic regression analysis.

Accurate prognostication of adverse pathological factors in localized prostate cancer is of utmost importance to ensure patients receive adequate pretreatment counseling. There are several therapeutic options particularly for low- and intermediate-risk disease, including prostatectomy and radiotherapy with or without ADT. In patients who opt for prostatectomy, however, the pathological specimen might show positive margins or pathological T3 disease, associated with a 13-27% and 40-50% probability of disease recurrence respectively (5, 24–26). Adjuvant radiotherapy for these patients improves metastasis-free survival but carries an increased risk of genitourinary and gastrointestinal toxicity (27). Salvage radiotherapy (i.e. initiated upon evidence of biochemical recurrence) may pose a safe alternative with only a 1 percentage point of change in 5-year event-free survival and a lower proportion of treatment related adverse events, as reported in a pre-planned meta-analysis (28) of three randomized trials studying these treatment options (7, 29, 30).

Since primary radiotherapy is another curative option for localized prostate cancer, it seems logical to optimize the selection of surgical candidates to reduce the necessity for multimodal therapy. However, when evaluated alongside other known prognostic factors such as pre-prostatectomy PSA and biopsy Gleason score, PTP-1B does not seem to be an independent prognostic factor. In line with this, the addition of the PTP-1B expression score to the PSA and Gleason did not significantly improve the sensitivity and specificity to predict the presence of APFs. Our sample was relatively small, and to detect an improvement over already available pre-prostatectomy prognostic factors, our experiment might need to be replicated in a larger cohort.

Hyperglycemia and other components of the metabolic syndrome have been associated with an increased prevalence of adverse pathological features and an increased risk biochemical recurrence of prostate cancer (31). Interestingly, in our study, PTP-1B expression was significantly associated with adverse pathological features, while the presence of diabetes mellitus and metformin usage were not. We did not collect data on pre-prostatectomy glucose levels but, given the low prevalence of insulin use in our study population, and that patients were fit for surgery, it would be expected that our study population had a low rate of uncontrolled hyperglycemia despite 20% of the population having a prior diagnosis of type 2 diabetes mellitus.

In this study, we found a weak positive correlation between PTP-1B expression and Gleason score, which might be related to PTP-1B expression in neuroendocrine cells of prostate cancer in humans, since the prevalence of neuroendocrine differentiation increases in tumors of higher grade (32). This supports the association between a higher PTP-1B expression score and poorer prognosis, despite PTP-1B expression not being an independent predictor for adverse pathological features. In addition, in recent years, there has been renewed interest in PTP-1B as a druggable target (33). A dual inhibitor of PTP-1B and TC-PTP has shown to induce anti-tumor immunity in animal models of cancer resistant to PD-1 blockade (34) and is currently being investigated in a phase 1 study of advanced solid tumors (ClinicalTrials.gov identifier NCT04777994). Therefore, as future directions for this study, we plan to construct this immunostaining panel, including immune-related markers and analyze its association with adverse pathological features and other outcomes.

Our survival analysis did not show a significant difference in OS or CSS. However, this study was underpowered for this analysis, and median OS and CSS were not reached despite a long follow-up, which is expected in localized prostate cancer. Replication in a larger cohort that also includes patients who received primary radiotherapy could perhaps provide deeper insights into the relationship between PTP-1B expression and long-term outcomes.

Quantification of expression of PTP-1B can be achieved with other methods, such as Western blot or PCR (19, 35). Nevertheless, we decided to use immunohistochemistry since it is a readily available technique, even in resource-limited settings. However, it is important to recognize that expression by IHC does not necessarily reflect the production of a functional protein. PTP-1B in its inactive form is ligated to the endoplasmic reticulum near the nucleus, and in the cytoplasm near the cell membrane in its active form. The location of the latter allows it to interact with the insulin receptor, and other receptor tyrosine kinases such as JAK2 and c-Src.

Due to the intricate relationships of intracellular signaling processes, an immunostaining panel including other markers implicated in migration, invasion and proliferation of prostate cancer (36) might improve on already described clinical markers. Potential candidates for such markers could be PTEN, RB1, NKX3, TP53, TMPRSS2, ATM, MYC, enolase, DLX2, Ki67, and estrogen receptor (37, 38). Loss of PTEN, higher expression of Ki67 and overexpression of MYC in high-risk prostate cancer prostatectomy specimens have been associated with worse progression-free survival (39).

In addition to the previously mentioned, we acknowledge as additional limitations of this study its retrospective design and the extended observation period which limited the availability and quality of archival prostatectomy specimens, which underscores the necessity for continued research.

In conclusion, PTP-1B expression, as determined by immunohistochemistry, was higher in patients with adverse pathological factors in prostatectomy specimens, independently of T2DM or prior metformin use. However, PTP-1B expression does not add additional prognostic value to previously known prognostic factors such as PSA and Gleason score.

## Data availability statement

The raw data supporting the conclusions of this article will be made available by the authors, without undue reservation.

## Ethics statement

The studies involving humans were approved by Instituto Nacional de Ciencias Medicas y Nutricion Salvador Zubiran Institutional Review Board. The studies were conducted in accordance with the local legislation and institutional requirements. The participants provided their written informed consent to participate in this study.

## Author contributions

MB: Conceptualization, Formal analysis, Funding acquisition, Investigation, Writing – original draft, Writing – review & editing. SU-R: Data curation, Investigation, Writing – review & editing. HV-A: Visualization, Writing – original draft, Writing – review & editing. MM-P: Investigation, Writing – original draft. HV: Investigation, Writing – review & editing. EL-R: Formal analysis, Writing – review & editing. YA-F: Formal analysis, Writing – review & editing. MD-G: Data curation, Formal analysis, Investigation, Writing – review & editing.
